# Supplementary material for: InterMEL: An international biorepository and clinical database to uncover predictors of survival in early-stage melanoma
Source: PLoS One. 2023 Apr 3;18(4):e0269324. doi: 10.1371/journal.pone.0269324 (PMC10069769; doi:10.1371/journal.pone.0269324)
Supplement: S1 Table — (PDF) [file pone.0269324.s001.pdf]

Supporting Table S1. InterMEL: Participants and melanoma variables and definitions

InterMEL Variable	Definition	Value type	Code	Required?	Notes	Responsible Data Source
**MELID**	Patient ID	Unique ID	IML-[Center Code]### or<br>IML-[Center Code]###N<br><br>Center Codes:<br>CCF = 1, CW = 2, DTM = 3, MDA= 4, MIA = 5, MSK = 6, NYU = 7, UNC = 8, Yal = 9	**Y**	Example:<br>IML-5001 or IML-5001N<br>The first number is the center code (IML-5001), the following 3 numbers are for the specific patient assigned in sequential order (IML-5001). An "N" at the end indicates Normal (IML-5001N).	Center PI sending tumors is responsible to make up the MELID, using IML-center number, patient number, and N or blank
**GROUP**	Comparison Group	Number Code	0 = Died from melanoma within 5 years of diagnosis<br>1 = Lived more than 5 years after diagnosis	**Y**		Center PI
**YEAR_DX**	Year of diagnosis of the first primary	Integer	YYYY	**Y**	Format YYYY	Center PI
**VITAL_STATUS**	Vital status at the last contact	Number Code	1 = Alive<br>2 = Dead of melanoma<br>3 = Dead of other causes<br>4 = Dead of unknown causes<br>99 = Unknown/ Missing	**Y**	Alive includes 'Alive w/ Melanoma' and 'Alive w/o Melanoma'<br>[Eligibility: Must have died within 5 years of diagnosis and those who lived more than 5 years after diagnosis without recurrence]	Center PI
**VITAL_STATUS_DATE**	The last follow up date or date of death	Date	MM/DD/YYYY	**Y**	If month unknown use '00' , if date unknown use '00', If entire date unknown type 'UNK'. Will be reconciled to '06' or '15' respectively during data cleaning.	Center PI
vital_status_source	Source of Vital status	Number Code	1 = Physician's Office/Private Medical Practitioner (LMD)<br>2 = Death Certificate (i.e. NDI)<br>3 = Other clinical information<br>99 = Unknown/ Missing			Center PI
vital_status_source_os	Specify other source	Text				
**FUDAYS**	Number of days from diagnosis to last follow-up	Integer	[CALCULATED]	**Y**	Calculated variable of YEARDX and VITALSTATUSDATE	Center PI
**MULT_PRIMARY**	Multiple primary of melanoma	Number Code	0 = No<br>1 = Yes<br>99 = Unknown/ Missing	**Y**	[Eligibility: The first primary invasive melanoma is eligible, without regard for subsequent melanomas, if synchronous select deeper]	Center PI
**AGE_AT_DX**	Age at diagnosis	Integer		**Y**		Center PI
**SEX**	Sex	Number Code	1 = Male<br>2 = Female<br>99 = Unknown/ Missing	**Y**	[Eligibility: Must equal 1 or 2, Unknown/ Missing not eligible]	Center PI
race	Race	Number Code	1 = White<br>2 = Asian<br>3 = American Indian/Alaska Native<br>4 = Black or African American<br>5 =Native Hawaiian or Other Pacific Islander<br>6 = More than one race<br>7 = Other<br>99 = Unknown/ Missing			Center PI

Supporting Table S1. InterMEL: Participants and melanoma variables and definitions

InterMEL Variable	Definition	Value type	Code	Required?	Notes	Responsible Data Source
ethnicity	Ethnicity (when possible)	Number Code	1 = Hispanic<br>2 = Non-Hispanic<br>99 = Unknown/ Missing			Center PI
**STAGE_AJCC8**	Pathological staging at time of diagnosis based on AJCC8	Number Code	1 = II<br>2 = IIA<br>3 = IIB<br>4 = IIC<br>5 = III<br>6 = IIIA<br>7 = IIIB<br>8 = IIIC<br>9 = IIID	**Y**	Note, with Breslow thickness, mitotic rate, and ulceration, we can assign T stage to any tumor using any system.[Eligibility: Tumors should be 1.00 mm (1-2.00 with ulceration) or more. No distant metastases at diagnosis.]. AJCC8 added microsatellites, microsatellites and in-transit mets to nodal status.	Center PI
stage_T_ajcc8	AJCC8 TNM staging - T (as available)	Number Code	0 = T0: No evidence of primary tumor/or site of primary tumor is unknown'<br>1 = T1: ≤ 1.0mm, unkwn ulceration [Not Eligible];<br>2 = T1a: <0.8mm, w/o ulceration [Not Eligible];<br>3 = T1b: <0.8mm, w/ ulceration or 0.8-1.0mm w/ or w/o ulceration [Not Eligible];<br>4 = T2: >1.0-2.0mm, unkwn ulceration;<br>5 = T2a: >1.0mm-2.0 mm, w/o ulceration;<br>6 = T2b: >1.0mm-2.0 mm, w/ ulceration;<br>7 = T3: >2.0-4.0mm, unkwn ulceration;<br>8 = T3a: >2.0mm-4.0mm, w/o ulceration;<br>9 = T3b: >2.0mm-4.0mm, w/ ulceration;<br>10 = T4: >4.0mm, unkwn ulceration<br>11 = T4a: >4.0 mm, w/o ulceration;<br>12 = T4b: > 4.0 mm, w/ ulceration;<br>13 = Tis: Melanoma in situ<br>98 = TX: Primary tumor thickness cannot be assessed<br>99 = Unknown/ Missing	**Y**	[Eligibility: Tumors should be 1.00 mm (1-2.00 with ulceration) or more.]	Center PI

Supporting Table S1. InterMEL: Participants and melanoma variables and definitions

InterMEL Variable	Definition	Value type	Code	Required?	Notes	Responsible Data Source
stage_N_ajcc8	AJCC8 TNM staging - N (as available)	Number Code	0 = N0: No spread to nearby lymph nodes<br>1 = N1: one tumor-involved node or any number of in-transit, satellite, and/or microsatellite metastases with no tumor-involved nodes<br>2 = N1a: One clinically occult (i.e., detected by SLN biopsy)<br>3 = N1b: one clinically detected<br>4 = N1c: no regional lymph node disease, but with presence of in-transit, satellite and/or microsatellite metastases<br>5 = N2: Two or three tumor-involved nodes or any number of i-transit, satellite, and/or microsatellite metastases with one tumor-involved node<br>6 = N2a: Two or three clinically occult (i.e., detected by SLN biopsy)<br>7 = N2b: Two or three, at least one of which was clinically detected<br>8 = N2c: One clinically occult or clinically detected LN, with the presence of in-transit, satellite, and/or microsatellite metastases<br>9 = N3: Four or more tumor-involved node or any number of in-transit, satellite, and/or microsatellite metastases with two or more tumor-involved nodes, or any number of matted nodes without or with in-transit, satellite, and/or microsatellite metastases<br>10 = N3a: Four or more clinically occult (i.e., detected by SLN biopsy)<br>11 = N3b: Four or more, at least one of which was clinically detected., or presence of any number of matted nodes<br>12 = N3c: Two or more clinically occult or clinically detected and/or presence of any number of matted nodes as well as presence of in-transit, satellite, and/or microsatellite metastases.<br>98 = NX: Nearby (regional) lymph nodes cannot be assessed<br>99 = Unknown/ Missing	**Y**	Provide as much information on Lymph node status (clinically palpable or through SLN) as possible. See SLN_status for neagtive/positive.	Center PI
stage_M_ajcc8	AJCC8 TNM staging - M (as available)	Number Code	0 = M0 - No distant metastasis<br>1 = M1a [Not Eligible]<br>2 = M1b [Not Eligible]<br>3 = M1c [Not Eligible]<br>4 = M1d [Not Eligible]<br>99 = Unknown/ Missing	**Y**	[Eligibility: M1/Stage IV not eligible]	Center PI
initial_breslow	Breslow Thickness (mm)	Number	[999 if Missing]			Center PI

Supporting Table S1. InterMEL: Participants and melanoma variables and definitions

InterMEL Variable	Definition	Value type	Code	Required?	Notes	Responsible Data Source
initial_ulceration	Presence of ulceration - yes/no	Number Code	1= Absent<br>2= Present<br>3 = Indeterminate<br>99 = Unknown/ Missing			Center PI
**PRIMARY_TUMOR_SITE**	Primary tumor location - body site (ICD-O-3 SITE CODES)	Number Code	0 = C44.0 Skin of lip, NOS<br>1 = C44.1 Eyelid<br>2 = C44.2 External ear<br>3 = C44.3 Skin of other and unspecified parts of face<br>4 = C44.4 Skin of scalp and neck<br>5 = C44.5 Skin of trunk<br>6 = C44.6 Skin of upper limb and shoulder<br>7 = C44.7 Skin of lower limb and hip (Including buttocks)<br>8 = C44.8 Overlapping lesion of skin<br>9 = C44.9 Skin, NOS<br>99 = Unknown/ Missing	**Y**	https://training.seer.cancer.gov/melanoma/abstract-code-stage/codes.html - we will make a note for the very rare cutaneous genital sites and code as C44.9, NOS	Center PI
tumor_burden	Size (mm) of size of the largest tumour deposit	Number	99 = Unknown/ Missing		Provide if possible. The recorded measurements should be the size of the largest tumour deposit (not the size of the involved lymph node, under less it is entirely replaced by tumour). (The size of the largest metastasis within sentinel lymph nodes is a strong predictor of the presence of additional positive non-sentinel lymph nodes within the lymph node basin and also a strong predictor of survival/outcome)	Center PI
SLN_status	SLN evaluation status	Number Code	1 = Negative<br>2 = Positive<br>99 = Unknown/ Missing		Result of sentinel lymph node biopsy or fine-needle aspiration. Result detail is in the field stage_N_ajcc8	Center PI
tumor_subtype	Tumor subtype (if known)	Number Code	1 = BRAF positive<br>2 = NRAS positive<br>3 = Both BRAF and NRAS<br>4 = Neither BRAF or NRAS<br>5 = Other mutation<br>98 = Not Assessed<br>99 = Unknown/ Missing			Center PI
tumor_subtype_os	Other Tumor subtype (specify)	Text				Center PI
progression	Evidence of progression - Progressive disease noted prior to or during first course of treatment	Number Code	1 = Yes<br>2 = No<br>99 = Unknown/ Missing		Based on NCI Definition: Becomes worse (stage) or spreads in the body (metastasizes)	Center PI
progression_date	Date of progression	Date	MM/DD/YYYY		If month unknown use '00' , if date unknown use '00', If entire date unknown type 'UNK'. Will be reconciled to '06' or '15' respectively during data cleaning.	Center PI

Supporting Table S1. InterMEL: Participants and melanoma variables and definitions

InterMEL Variable	Definition	Value type	Code	Required?	Notes	Responsible Data Source
recurrence	Recurrence - After curative treatment and an observed disease free-interval period (see additional definition in notes)	Number Code	1 = Yes<br>2 = No<br>99 = Unknown/ Missing		Based on NCI Definition: Melanoma that has recurred (come back), usually after a period of time during which the melanoma could not be detected. The melanoma may come back to the same place as the original (primary) tumor or to another site. However, the stage will not have changed and there will be no organ metastases.	Center PI
recurrence_date	Recurrence date (if known)	Date	MM/DD/YYYY		If month unknown use '00' , if date unknown use '00', If entire date unknown type 'UNK'. Will be reconciled to '06' or '15' respectively during data cleaning.	Center PI
recurrence_site	Anatomic site or recurrence	Text	[Describe the site of recurrence]			Center PI
AntiMEK_inhibitor	Treatment with Vemurafenib, Dabrafenib Cobimettinib, trametinib?	Number Code	1 = Yes<br>2 = No<br>99 = Unknown/ Missing			Center PI
AntiMEK_inhibitor_date	Treatment date if known	Date	MM/DD/YYYY		If month unknown use '00' , if date unknown use '00', If entire date unknown type 'UNK'. Will be reconciled to '06' or '15' respectively during data cleaning.	Center PI
iIpilimumab	Treatment with Ipilimumab?	Number Code	1 = Yes<br>2 = No<br>99 = Unknown/ Missing			Center PI
iIpilimumab_date	Treatment date if known	Date	MM/DD/YYYY		If month unknown use '00' , if date unknown use '00', If entire date unknown type 'UNK'. Will be reconciled to '06' or '15' respectively during data cleaning.	Center PI
antiPD_1	Treatment with Anti-PD1 (Nivolumab, pembrolizumab)?	Number Code	1 = Yes<br>2 = No<br>99 = Unknown/ Missing			Center PI
antiPD_1_date	Treatment date if known	Date	MM/DD/YYYY		If month unknown use '00' , if date unknown use '00', If entire date unknown type 'UNK'. Will be reconciled to '06' or '15' respectively during data cleaning.	Center PI
interferon	Treatment with Interferon?	Number Code	1 = Yes<br>2 = No<br>99 = Unknown/ Missing			Center PI
interferon_date	Treatment date if known	Date	MM/DD/YYYY		If month unknown use '00' , if date unknown use '00', If entire date unknown type 'UNK'. Will be reconciled to '06' or '15' respectively during data cleaning.	Center PI
other_treatment	Other Treatment	Number Code	1 = Yes<br>2 = No<br>99 = Unknown/ Missing			Center PI
other_treatment_os	Other Treatment (specify)	Text				Center PI

Supporting Table S1. InterMEL: Participants and melanoma variables and definitions

InterMEL Variable	Definition	Value type	Code	Required?	Notes	Responsible Data Source
other_treatment_date	Treatment date if known	Date	MM/DD/YYYY		If month unknown use '00' , if date unknown use '00', If entire date unknown type 'UNK'. Will be reconciled to '06' or '15' respectively during data cleaning.	Center PI
height	Height at diagnosis	Number	999 = Unknown/ Missing		Inches or Centimeters ONLY	Center PI
height_unit	Units for Height at Dx	Number Code	1 = Inches (in)<br>2 = Centimeters (cm)		Specify Units	Center PI
weight	Weight at diagnosis	Number	999 = Unknown/ Missing		Pounds or Kilograms ONLY<br>(NOTE: Missing is set to 999 due to biologically plausible value for 99kg)	Center PI
weight_unit	Units for Weight at Dx	Number Code	1 = Pounds (lb)<br>2 = Kilograms (kg)		Specify Units	Center PI
bmi	BMI (kg/m^2) at diagnosis	Number	99 = Unknown/ Missing		Calculated variable using HEIGHT and WEIGHT	Center PI
center_notes	Additional notes from Center	Note Field				Center PI
path_id	Pathology Record [Auto Populated]	Number			Automatically Generated	Dermatopathologist/Core 2 via REDCap Entry System
path_date	Date Assessed	Date	MM/DD/YYYY			Dermatopathologist/Core 2 via REDCap Entry System
path_initials	Pathologist Initials	Number Code	1 = KB<br>2 = CL			Dermatopathologist/Core 2 via REDCap Entry System
specimen	Specimen Type	Number Code	1 = Biopsy<br>2 = Excision			Dermatopathologist/Core 2 via REDCap Entry System
hist	Histology (ICD-O-3 Morphology Codes)	Number Code	1 = Malignant Melanoma, NOS (8720/3)<br>2 = Nodular Melanoma (8721/3)<br>3 = Malignant Melanoma, Regressing (8723/3)<br>4 = Amelanotic Melanoma (8730/3)<br>5 = Lentigo Maligna Melanoma (8742/3)<br>6 = Superficial Spreading Melanoma (8743/3)<br>7 = Acral Lentiginous Melanoma (8744/3)<br>8 = Desmoplastic Melanoma (8745/3)<br>9 = Mixed<br>10 = Other, Specify<br>99 = Unknown/ Missing		https://training.seer.cancer.gov/melanoma/abstract-code-stage/morphology.html	Dermatopathologist/Core 2 via REDCap Entry System
hist_os	Histology - Other, Specify	Text			Descibe other histologic code	Dermatopathologist/Core 2 via REDCap Entry System
thickness	Breslow Thickness (mm)	Number	[999 if Missing]			Dermatopathologist/Core 2 via REDCap Entry System
clarklevel	Clark Level	Number Code	1 = I<br>2 = II<br>3 = III<br>4 = IV<br>5 = V			Dermatopathologist/Core 2 via REDCap Entry System
mitoses	Presence of mitoses?	Number Code	0 = Absent<br>1 = Present<br>99 = Cannot assess			Dermatopathologist/Core 2 via REDCap Entry System
mit_index_mm	Present Mitotic Index (#/mm2)	Number				Dermatopathologist/Core 2 via REDCap Entry System
mit_index_na	Flag for #/slide	Number Code	1 = Lesion area does not permit assessment per mm2			Dermatopathologist/Core 2 via REDCap Entry System

Supporting Table S1. InterMEL: Participants and melanoma variables and definitions

InterMEL Variable	Definition	Value type	Code	Required?	Notes	Responsible Data Source
mit_index_slide	Mitotic Index (#/slide)	Number				Dermatopathologist/Core 2 via REDCap Entry System
ulceration	Presence of ulceration - yes/no	Number Code	0 = Absent<br>1 = Present<br>3 = Indeterminate<br>99 = Cannot Assess			Dermatopathologist/Core 2 via REDCap Entry System
TILs	Tumor infiltrating lymphocytes	Number Code	1 = Brisk<br>2 = Non-brisk<br>3 = Absent<br>99 = Cannot assess			Dermatopathologist/Core 2 via REDCap Entry System
regression	Regression	Number Code	0 = Absent<br>1 = Present<br>99 = Cannot assess			Dermatopathologist/Core 2 via REDCap Entry System
regression_loc	Regression - Present	Number Code	1 = Papillary dermis<br>2 = Reticular dermis			Dermatopathologist/Core 2 via REDCap Entry System
T_pigmentation_type	Presence of melanin pigment	Number Code	0 = Absent<br>1 = Present Lightly pigmented<br>2 = Present, Heavily pigmented<br>99 = Cannot assess			Dermatopathologist/Core 2 via REDCap Entry System
T_pigmentation	Pigmentation - Present (may specify, e.g., pigmented epithelioid cells or amelanotic spindle cells)	Text				Dermatopathologist/Core 2 via REDCap Entry System
T_nevus	Presence of associated nevus	Number Code	0 = Absent<br>1 = Present<br>99 = Cannot assess			Dermatopathologist/Core 2 via REDCap Entry System
satellites	Satellites	Number Code	0 = Absent<br>1 = Present<br>2 = Not applicable (Biopsy only)<br>99 = Cannot assess			Dermatopathologist/Core 2 via REDCap Entry System
se	Solar Elastosis	Number Code	0 = Absent<br>1 = Mild/Moderate<br>2 = Severe<br>88 = Cannot assess<br>99 = Unknown/ Missing			Dermatopathologist/Core 2 via REDCap Entry System
cell_type	Cell Type	Text				Dermatopathologist/Core 2 via REDCap Entry System
purity	Purity	Text				Dermatopathologist/Core 2 via REDCap Entry System
pni	Perineural Invasion (PNI)	Number Code	1 = Present<br>0 = Absent		Added 1/23	Dermatopathologist/Core 2 via REDCap Entry System
path_notes	Pathology Assessment Notes	Note Field			Any additional notes	Dermatopathologist/Core 2 via REDCap Entry System
