# Supplementary material for: InterMEL: An international biorepository and clinical database to uncover predictors of survival in early-stage melanoma
Source: PLoS One. 2023 Apr 3;18(4):e0269324. doi: 10.1371/journal.pone.0269324 (PMC10069769; doi:10.1371/journal.pone.0269324)
Supplement: S1 Appendix — (DOCX) [file pone.0269324.s002.docx]

**Appendix 1: Methods**

***Overview of the Biospecimens Core workflow.*** As depicted in **Figure 1**, the Biospecimen Core (1) receives unstained FFPE tissue sections (for co-extraction of nucleic acids, and IHC) and H&E stained slides (for histopathologic review) from all the contributing centers; (2) conducts the initial histopathologic review to classify cases into qualifying/non-qualifying tissues for nucleic acid extraction, assess tumor quantity and purity, and guide the macro/microdissection of tumor areas for nucleic acids co-extraction and non-tumor DNA extraction. H&Es are mailed to UNC for Aperio scanning and returned to MSK for a second, detailed histopathologic review; (3) extracts DNA and RNA from tumor tissue and DNA from normal tissue; characterizes the extracted nucleic acids using quantity, purity and integrity measures.(4) Distributes DNA and RNA specimens for testing: DNAs to the MSK iGO core for testing of somatic mutations with the MSK-IMPACTTM assay (for Research Project 1), DNAs to UNC for analyses of methylation (Research Project 2), and RNAs to NYU for analysis of miRNA expression (Research Project 3). Unstained tissue sections are mailed to UNC for phenotypic assessments (Research Projects 1 and 2).

***Procurement of archival tissue.*** To provide adequate amount of tissue for genetic and non-genetic downstream testing, estimate the dimensions of the melanoma tumor considering the area that will likely qualify for the extraction of nucleic acids. Multiply this estimated area by the number of planned 10µm sections to obtain the estimated combine area. If the combined area is <500mm^2^, whenever feasible, plan to procure additional 10µm tissue sections on uncharged slides. Avoid tissues that were treated with decalcifying solutions (for removal of bone). For non-tumor matching tissue samples obtained from wide-excisions, lymph nodes, or other organs, tissue must be free of tumor cells and viable (no necrosis or scarring upon inspection of H&E), avoiding adipose tissue. Centers can provide 200ng of double stranded germline DNA (dsDNA), frozen whole blood, buffy coat, or saliva.

To minimize potential cross contamination of tissues and derived nucleic acids, sectioning labs are advised to use disposable blades, change blades between blocks, and change the water in the basin used to spread sections onto slides.

For tumor FFPEs, five to fifteen sections of 10µm thickness are placed on uncharged slides, two thin (4 or 5µm) unstained sections on charged slides, and two additional thin (4 or 5µm) sections (first and last) placed on charged slides are stained with hematoxylin-eosin (H&E) and covered. Primary melanomas are generally very small (i.e., few mm) compared to other types of tumors, for example breast cancer (2-5cm), therefore additional guidance is provided to the participating centers to increase the likelihood of obtaining sufficient nucleic acids, as shown in the Supporting Material. For non-tumor matching archival tissues (e.g., surrounding skin), at least one H&E is requested to confirm absence of tumor cells, scarring, etc. Centers can opt to provide 200 ng of double stranded germline DNA (dsDNA), frozen whole blood, buffy coat, saliva, or solid tissue including skin from wide excisions or tissue from negative lymph nodes.

Participating centers are also advised to ship the sections to MSK as soon as possible after tissues are sectioned, and to provide information on the sectioning date(s) or time elapsed between sectioning and shipping. Upon arrival to the MSK’s Molecular Epidemiology Laboratory, lab members proceed to: (i) capture the information in a Specimen Log, (ii) fill out a Pathology sample manifest for the H&Es received and distribute these to our reference pathologists for initial review, and (iii) fill out a shipping manifest for thin unstained sections and prepare these for shipping to UNC (overnight at ambient temperature). These unstained thin sections are saved for future TIL and TME assessment. Upon arrival to UNC, thin unstained sections are stored in a nitrogen gas-filled cabinet to reduce oxidation prior to downstream staining.

***Optimization of RNA-DNA extraction from FFPE tissue.*** To evaluate the methods and logistics involved for the isolation of nucleic acids from a common cell lysate, we first used an independent (pilot) set of 34 FFPE tumor and 23 non-tumor tissues (8 to 10 sections each, 10µm thickness). For 14 of the tumor sets, sections were divided into two and used to compare two RNA and DNA co-extraction kits: the ALLPrep DNA/RNA FFPE kit (Qiagen) or ‘co-extraction kit A’ and the FFPE RNA/DNA Purification Plus Kit (Norgen) or ‘co-extraction kit B’. Twenty additional sets of tissue sections were extracted with co-extraction kit A, for optimization. For 6 of the non-tumor sets, sections were similarly divided into two and used to compare the QIAamp DNA FFPE Tissue Kit or ‘DNA extraction kit A’, and the FFPE DNA Isolation Kit or ‘DNA extraction kit B’. Sixteen additional sets of tissue sections were used to optimize the extraction while using the DNA extraction kit A. Manufacturers’ protocols were followed, and then columns were eluted a second time to recover additional material in a separate tube. To determine the kit and method of preference we compared the quantity and quality parameters of both RNA and DNA, as well as hands-on time and overall workflow.

***Histopathology-guided co-extraction from archival tissues: current study.*** For each tumor specimen, systematic marking of qualifying tissue areas on the reverse of each of the unstained 10µm FFPE tissue sections mounted on uncharged slides is performed in the Molecular Epidemiology Laboratory at MSK. This is followed by careful scraping of tissues, and then co-extraction of nucleic acids, in batches. Visualization of tissue demarcation is aided by a lit magnifying lens and a humidifier as needed. Based on preliminary evaluation on an independent set of pilot specimens and comparison of two commercial kits (see above) co-extractions of DNA and RNA from tumor FFPE tissue are done with the AllPrep® DNA/RNA FFPE Kit (Qiagen). Manufacturer’s recommendations were followed with: (i) deparaffinization solution utilized as solvent; (ii) DNA pellets obtained on day 1 are washed with PBS before adding proteinase K and incubated overnight in a thermo-shaker at 56ºC and 300rpm; (iii) DNA is incubated at 90ºC for one hour; (iv) RNA and DNA are incubated for 5 minutes before completing the elution, and (v) a second elution is obtained in a separate tube after incubating the sample for 2 minutes.

***Aliquoting of nucleic acids.*** Samples are aliquoted according to pre-determined rules. For the first RNA eluate, 5µL are reserved for same- day QC measures. A second and third aliquot contain a minimum of 4µL of RNA containing a minimum of 500ng and are reserved for testing, leaving the remaining RNA in the original tube. RNA from the second elution is used for QC and the remaining kept for later use in the original tube. For the DNA eluate, 5µL are reserved for QC measures, as described next. DNA and RNA samples are stored in Eppendorf tubes at -80°C.

***Extraction of germline DNA (gDNA).*** DNAs are extracted from FFPE tissue sections and curls with the QIAmp DNA FFPE Tissue Kit, scraping up to a combined area of 2000mm^2^ per tube, and applying 320µL of deparaffinization solution per 600mm2 of tissue. gDNAs is eluted twice using 30µL and 20µL ATE buffer, to obtain the 1^st^ and 2^nd^ elution. For frozen blood or PBMC, DNAs are extracted with QiAmp DNA Blood Mini Kit or FlexiGene DNA Kit (250) following manufacturer’s protocols. Buccal samples collected with the OGR-500 saliva collection kit (Genotek, Ottawa, Canada) are extracted with PrepIT-L2P and following the manufacturer’s procedures.

***Assessment of DNA and RNA quantity and quality***. DNA and RNA samples are quantified using the Nanodrop^TM^ (ND8000) and Qubit dsDNA Broad Range Assay. RNA samples are further assessed for quantity and quality on a TapeStation 2200 (Agilent) to assess quantity and proportion of RNA of >200nt in length (quality matrix DV200). DNAs are also evaluated on pre-made 2% agarose gels (Invitrogen) to assess degree of fragmentation, ‘amplifiability’ potential for amplicons of up to 400bp through a multiplex QC-PCR [van Beers et al. 2006], and to uncover or rule out potential cross-contamination or mislabeling/identity mix-up at any point before or during the study within tumor-normal DNA pairs or aliquots. For this, we use the SampleID assay (Agena Bioscience) which targets 44 highly prevalent SNPs and 3 biological sex markers. We look for overall typing success, and for agreements between self-reported and genetic sex, and within paired normal-tumor DNAs. Providing centers are informed of mismatched sex and/or tumor-normal DNA pairs, and new samples are requested when necessary. Tissue and derived samples labelled with the resistant 2D barcode-label are scanned throughout the processing and distribution for testing (iGO core for MSK-IMPACT^TM^ assay, UNC for methylation and IHC, and NYU for miRNA expression). Data elements are entered in batches, and quality control is maintained by a system of automatic edit checks that occur as the data are being entered. In addition, data pertinent to tissue specimens and derived nucleic acids are evaluated through frequency tables, monthly or bi-monthly. Summaries are generated for the nucleic acid output variables to examine the distributions and trends of specimen-related measurements, including DNA and RNA yields, and measurements of nucleic acids quality. Descriptive statistics include means, standard deviations, and medians for continuous measurements and frequencies and percentages for categorical variables. All analyses are conducted using R (R Foundation for Statistical Computing, Vienna, Austria).

***Screening of somatic mutations.*** At Memorial Sloan Kettering Cancer enter, DNA samples were submitted for sequencing with the Integrated Mutation Profiling of Actionable Cancer Targets or MSK-IMPACT™, a clinically validated and FDA approved hybridization capture-based next-generation sequencing assay developed to guide cancer treatment [Chen et al. 2015, Zehir et al. 2017]. Briefly, MSK-IMPACT^TM^ allows the identification of tumor mutations through targeted deep sequencing by capturing all protein-coding exons and select introns of up to 505 commonly implicated oncogenes, tumor suppressor genes, and members of pathways deemed actionable by targeted therapies. This assay obtains much higher coverage than the coverage obtained by whole exome or whole genome sequencing [Cheng et al. 2015]. For this ongoing study, after quantitation using the Quant-iT PicoGreen dsDNA Assay (ThermoFisher), 0.5-200ng of DNA are used to prepare libraries using the KAPA Hyper Prep Kit (Kapa Biosystems) with 8-9 cycles of PCR. Two to 196ng of each barcoded library are captured by hybridization in equimolar pools of 7-27 samples using the MSK-IMPACT™ assay (Nimblegen SeqCap), plus, for the current InterMEL project additional baits are used specific to the MC1R (melanocortin 1 receptor) gene. Captured pools were sequenced on a NovaSeq 6000, HiSeq 2500 in Rapid Mode, or HiSeq 4000 in a PE100 run using the NovaSeq 6000 S2 or S4 Reagent Kit (200 Cycles), HiSeq Rapid SBS Kit v2, or HiSeq 3000/4000 SBS Kit (Illumina), respectively, producing an average coverage of 241X per sample. The MSK-IMPACTTM assay modifications overtime have been tested and validated for clinical testing (<https://www.accessdata.fda.gov/cdrh_docs/reviews/den170058.pdf>); no adverse effects are anticipated for the research samples.

***Methylation profiling.*** At the University of North Carolina, 250-350ng of tumor dsDNA plus internal controls were treated with sodium bisulfite using the EZ DNA Methylation Gold kit (Zymo Research) according to the manufacturer’s instructions. Bisulfite modified DNAs were quantified with a NanodropTM spectrophotometer and stored at -20ºC until the next step. To improve the DNA, template bisulfite-modified DNAs were treated with the Illumina Infinium HD FFPE DNA Restore kit following manufacturer’s instructions. Genome-wide DNA methylation analysis at 866,895 CpG sites is performed using the Infinium MethylationEPIC BeadChip kit (Illumina) and the Illumina iScan system at the UNC Mammalian Genotyping core. MethylationEPIC data are assembled with the GenomeStudio Methylation module (v2.0). All batches include methylated and non-methylated DNA, both human (Zymo Research), and cell lines (CRL-3230, HTB-22; ATCC) to identify and control for potential batch effects. Each data point represents fluorescent signals from both methylated (Cy5) and unmethylated (Cy3) alleles. After subtracting background, for each interrogated CpG site, the methylation level is reported by a beta (β)-value, or ratio of fluorescence corresponding to the [(methylated allele)/(methylated + unmethylated alleles)], and calculated as β = max(Cy5,0)/(|Cy5|+|Cy3|+100). β values range from 0 (completely unmethylated) to 1.0 (fully methylated). Probes that either (a) map to more than one chromosomal location and probes overlapping a single nucleotide polymorphism, (b) have missing values, (c) have detection p-values >0.05 in over 30% of the samples (poor performing probes), and/or (d) map on the X and Y chromosomes are removed or masked. Single-sample (ss) Noob-normalization was applied to the β values followed by Beta Mixture Quantile (BMIQ) normalization. Methylation β values [raw β or beta mixture quantile dilation (BMIQ)-normalized and single-sample Noob-normalized] for individual CpGs were compared for array batch and study center using principal component analysis. Filtering and normalization of output methylation data obtained from the Infinium MethylationEPIC arrays is done in R [The R Project for Statistical Computing 2022].

***Assessment of miRNA expression on tumor RNA.*** RNA quality and quantity were analyzed on a Bioanalyzer 2100 (Agilent) using a pico chip, and aliquots with a minimum of 450ng of total RNA, regardless of RNA Integrity Number (RIN) or the quality matrix DV200 values, were distributed. For miRNA expression, 100ng of RNA in a volume of 3µL (maximum 5µL) were hybridized using the Nanostring nCounter® Human v3 miRNA Expression Assay, which includes 800 microRNAs. Five samples were used as inter-batch duplicates and as 4 to 5 repeats. All steps were performed according to the manufacturer’s protocol using the nCounter® MAX/FLEX System. Briefly, reactions were incubated with capture and reporter probes at 65ºC for over 16hrs / overnight in a PCR machine. Samples were bound to the chip on the Nanostring prep station for 3 hours. Upon completion, cartridges were read on the Nanostring Digital Analyzer using the high setting or the 280 fields of view option. Data were analyzed with Nanostring nSolver 2.0, and output consisted of a code set specific library file, raw counts and normalized datafiles. Raw counts/reads are normalized to positive controls and housekeeping genes using the nSOlver software. Samples with low miRNA content and/or proportion of probes above the minimal threshold, set at 10% for each run, were flagged. The background threshold was determined as the mean plus two standard deviations of the medians of the negative controls for each sample in a particular run.

***Data handling***. Eligibility is communicated to the tissue procurement centers, which triggers the distribution of pre-printed labels and processing of tissues and derived data as described earlier under Biospecimens (**Figure 1**). Pathology data are entered into a REDCap database where they are curated and added to the clinical information provided by the tissue contributing centers. Demographic, clinical, and pathologic data are distributed to investigative teams leading each of the scientific projects. Raw and processed data files from individual study centers are received, processed, and stored in six terabytes of dedicated disk space. To ensure that files were not corrupted during transmission we have used Globus.org file-sharing system. Data from different batches are stored separately. MSK-IMPACT™ files included Binary Alignment Map files, Variant Call Format, Fraction and Allelic Copy number Estimation from Tumor/normal Sequencing and QC files. Nanostring related files have been described (see above). All processed data were accompanied by descriptions on how data processing, normalization, background subtraction and adjustment for batch effect was done.

***Data Availability:*** All the data underlying this report can be accessed through the “InterMEL biorepository and clinical database to report methods & best practices_dataset-I” (1), “InterMEL biorepository and clinical database to report methods & best practices_dataset-II” (2), and “InterMEL biorepository and clinical database to report methods & best practices_dataset-III” (3), available through the Harvard Dataverse Network (<https://doi.org/10.7910/DVN/QG5KSR>, <https://doi.org/10.7910/DVN/HK7LVX>, and https://doi.org/10.7910/DVN/GD8UZG). Additional data will become available through the National Cancer Institute dbGaP website (accession 48808) once the embargo is lifted on December 1^st^, 2023. For additional information and/or data sharing requests contact [crdatashare@mskcc.org](mailto:crdatashare@mskcc.org).

***References:***

Cheng DT, Mitchell TN, Zehir A, Shah RH, Benayed R, Syed A, et al. Memorial Sloan Kettering-Integrated Mutation Profiling of Actionable Cancer Targets (MSK-IMPACT): A Hybridization Capture-Based Next-Generation Sequencing Clinical Assay for Solid Tumor Molecular Oncology. J Mol Diagn. 2015;17(3):251-64.

Zehir A, Benayed R, Shah RH, Syed A, Middha S, Kim HR, et al. Mutational landscape of metastatic cancer revealed from prospective clinical sequencing of 10,000 patients. Nat Med. 2017;23(6):703-13.

The R Project for Statistical Computing 2022 [Available from: [www.R-project.org](file:///\\VPensBST\BstShared\EpiLab\MOL.%20EPI.%20LAB%20S-732\TEMP_Manuscript%20grants%20etc\2022_IML%20Manuscript%20items_Irene-Alex\REVISION%20MATERIAL%20Sept%202022\www.R-project.org).]

Irene Orlow, Jessica M. Kenney, Kelli O’Connell, Keimya D. Sadeghi, Tim K. Lee, Cecilia Lezcano, Klaus J. Busam, Eva Hernando, Sharon N. Edmiston, Tawny W. Boyce, Christopher I. Amos, James S. Wilmott, Anne E. Cust, Richard A. Scolyer, Graham J. Mann, Hazel Burke, Valerie Jakrot, Ping Shang, Peter M. Ferguson, Jennifer S. Ko, Peter Ngo, Pauline Funchain, Judy R. Rees, Honglin Hao, Eloise Parrish,, Kathleen Conway, Paul B. Googe, David W. Ollila, Stergios J. Moschos, Douglas Hanniford, Diana Argibay, Jeffrey E. Lee, Iman Osman, Li Luo, Pei-Fen Kuan, Arshi Aurora, Bonnie E. Gould Rothberg, Marcus W. Bosenberg, Meg R. Gerstenblith, Cheryl Thompson, Paul N. Bogner, Ivan P. Gorlov, Sheri L. Holmen, Elise K. Brunsgaard, Yvonne M. Saenger, Ronglai Shen, Venkatraman Seshan, Eduardo Nagore, Marc S. Ernstoff, Colin B. Begg, Nancy E. Thomas, Marianne Berwick on behalf of the InterMEL Consortium, 2023, "InterMEL biorepository and clinical database to report methods & best practices_dataset-I", Harvard Dataverse, V1, <https://doi.org/10.7910/DVN/QG5KSR>.

Irene Orlow, Cecilia Lezcano, Klaus J. Busam, Keimya D. Sadeghi, Jessica M. Kenney, Kelli O’Connell, Tawny W. Boyce, Eva Hernando, Sharon N. Edmiston, Christopher I. Amos, James S. Wilmott, Anne E. Cust, Richard A. Scolyer, Graham J. Mann, Hazel Burke, Valerie Jakrot, Ping Shang, Peter M. Ferguson, Jennifer S. Ko, Peter Ngo, Pauline Funchain, Judy R. Rees, Honglin Hao, Eloise Parrish, Kathleen Conway, Paul B. Googe, David W. Ollila, Stergios J. Moschos, Douglas Hanniford, Diana Argibay, Jeffrey E. Lee, Iman Osman, Li Luo, Pei-Fen Kuan, Arshi Aurora, Bonnie E. Gould Rothberg, Marcus W. Bosenberg, Meg R. Gerstenblith, Cheryl Thompson, Paul N. Bogner, Ivan P. Gorlov, Sheri L. Holmen, Elise K. Brunsgaard, Yvonne M. Saenger, Ronglai Shen, Venkatraman Seshan, Eduardo Nagore, Marc S. Ernstoff, Colin B. Begg, Nancy E. Thomas, Marianne Berwick on behalf of the InterMEL Consortium, 2023, "InterMEL biorepository and clinical database to report methods & best practices_dataset-II", Harvard Dataverse, V1, <https://doi.org/10.7910/DVN/HK7LVX>.

Irene Orlow, Jessica M. Kenney, Keimya D. Sadeghi, Kelli O’Connell, Tim K. Lee, Cecilia Lezcano, Klaus J. Busam, Tawny W. Boyce, Eva Hernando, Sharon N. Edmiston, Christopher I. Amos, James S. Wilmott, Anne E. Cust, Richard A. Scolyer, Graham J. Mann, Hazel Burke, Valerie Jakrot, Ping Shang, Peter M. Ferguson, Jennifer S. Ko, Peter Ngo, Pauline Funchain, Judy R. Rees, Honglin Hao, Eloise Parrish, Kathleen Conway, Paul B. Googe, David W. Ollila, Stergios J. Moschos, Douglas Hanniford, Diana Argibay, Jeffrey E. Lee, Iman Osman, Li Luo, Pei-Fen Kuan, Arshi Aurora, Bonnie E. Gould Rothberg, Marcus W. Bosenberg, Meg R. Gerstenblith, Cheryl Thompson, Paul N. Bogner, Ivan P. Gorlov, Sheri L. Holmen, Elise K. Brunsgaard, Yvonne M. Saenger, Ronglai Shen, Venkatraman Seshan, Eduardo Nagore, Marc S. Ernstoff, Colin B. Begg, Nancy E. Thomas, Marianne Berwick on behalf of the InterMEL Consortium, 2023, "InterMEL biorepository and clinical database to report methods & best practices_dataset-III", Harvard Dataverse, V1, <https://doi.org/10.7910/DVN/GD8UZG>.
