# Supplementary material for: InterMEL: An international biorepository and clinical database to uncover predictors of survival in early-stage melanoma
Source: PLoS One. 2023 Apr 3;18(4):e0269324. doi: 10.1371/journal.pone.0269324 (PMC10069769; doi:10.1371/journal.pone.0269324)
Supplement: S2 Appendix — (PDF) [file pone.0269324.s003.pdf]

Appendix 2: Preliminary Results

Comparison of RNA/DNA co-extraction kits. The extraction procedures for co-extracting nucleic acids were very similar. Preliminary assessment of the quantities and quality of nucleic acids obtained using two commercially available RNA/DNA co-extraction kits and an unrelated set of FFPE melanomas (with sections split equally for use with each of the kits), revealed an average of 6.3µg totDNA, 3.6µg dsDNA, 15.2µg totRNA obtained with the co-extraction kit A. None of the RNAs had DV200 below 30%, indicating that at least 30% of the RNA strands were longer than 200nt. With the co-extraction kit B we obtained an average of 5.5µg totDNA, 1.4µg dsDNA, and 9.9µg totRNA; and 8% of the RNAs had DV200 below 30%. The number of bands amplified in the multiplex QC-PCR suggested a better amplifiability for co-extraction kit B: 13 (92%) of the DNAs amplified two or more bands, compared to 9 (64%) of the 'kit A' co-extracted DNAs. There was no difference in the degree of RNA fragmentation measured by the RNA Integrity Numbers (RIN) (1.80 vs. 1.85).

Assessment of miRNA/mRNA expression and mutation screening using co-extracted nucleic acids. Using the pilot set of co-extracted specimens, we evaluated RNA by screening miRNA expression and a custom mRNA Nanostring panel. After initial normalization, housekeeping genes signals were well above the calculated background across all groups and had very low CVs indicating consistency across the samples**.** Figure S2 shows clustered values for 12 samples (corresponding to three melanoma tumors, two co-extraction kits, and first and second elutions), using log2 mean centered intensities. The first cluster (left) represents the full dataset, while low signals ($\leq$50) were removed in the second cluster (right). No differences were noticed by extraction kit or elution, and the scatter is observed only for the low intensity points. Two randomly selected paired tumor-normal DNA samples were screened for mutations with the MSK-IMPACT™ assay, for the evaluation of resulting libraries, and coverage upon NGS. We obtained libraries with concentrations ranging between 15.7 to 28.34ng/µl (very good, if >11ng; good if 8-10ng/µl, and poor if < 4ng/µl). The median coverage for the tumors was 362x with no evidence of QC issues.
