# Supplementary material for: InterMEL: An international biorepository and clinical database to uncover predictors of survival in early-stage melanoma
Source: PLoS One. 2023 Apr 3;18(4):e0269324. doi: 10.1371/journal.pone.0269324 (PMC10069769; doi:10.1371/journal.pone.0269324)
Supplement: S3 Appendix — S1 Fig. Quantity and quality assessment of RNA and DNA co-extracted from FFPE. S2 Fig. Performance of RNA co-extraction kit and elution on NanostringTM. S3 Fig. Effect of FFPE tissue input on the yield of co-extracted nucleic acids. S4 Fig. Effect of TILs and pigmentation on DNA purity and amplifiability. S5 Fig. Effect of the DNA and RNA quality on recommendations to proceed with synthesis of libraries and NGS/MSK-IMPACTTM. S6 Fig. Comparison of DNA- and library-QC recommendations with coverage obtained in melanomas screened with the MSK-IMPACTTM assay. (PDF) [file pone.0269324.s004.pdf]

Appendix 3: Supporting Figures

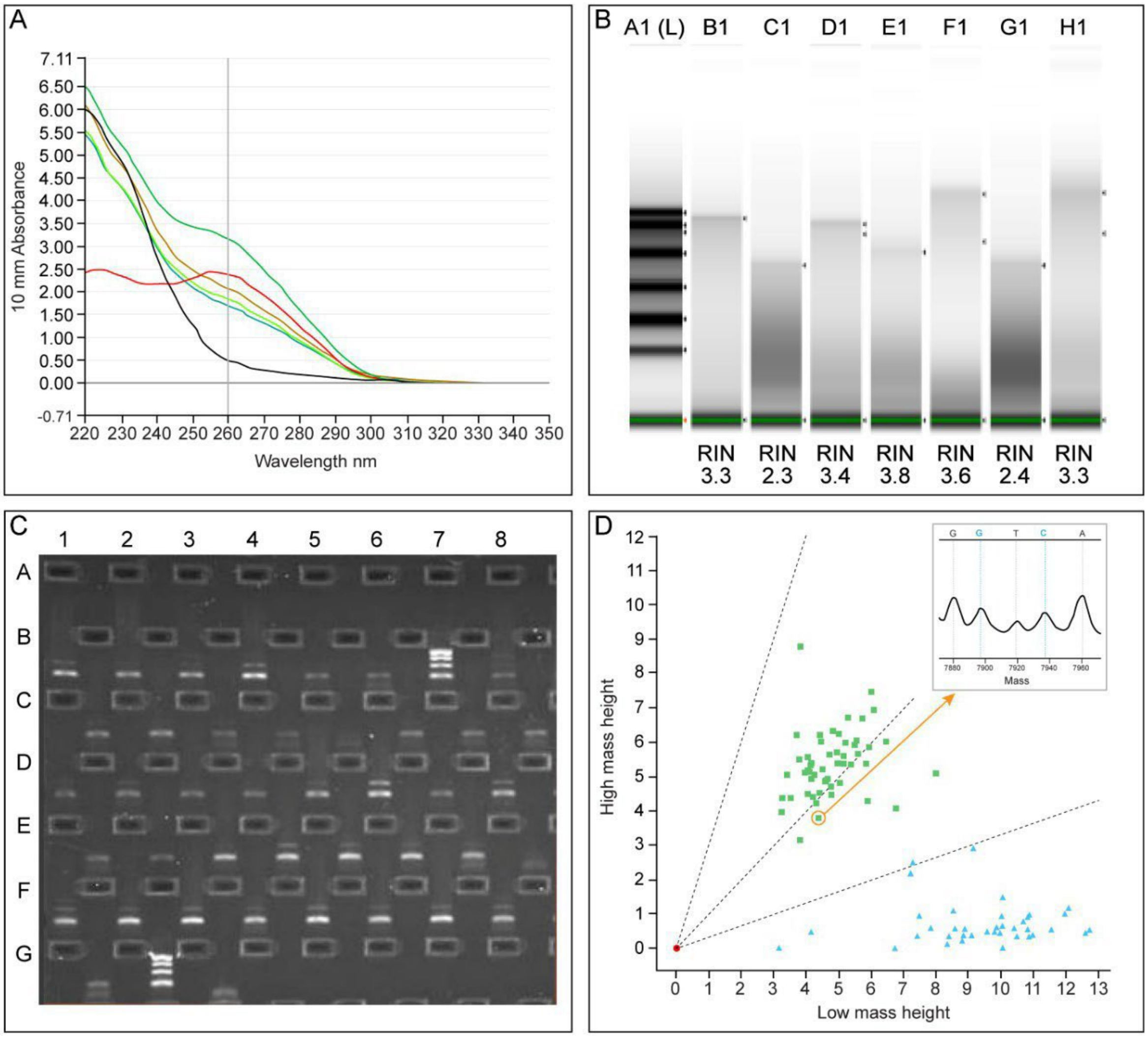

Figure S1. Quantity and quality assessment of RNA and DNA co-extracted from FFPE. (A) Total DNA is quantified with a Nanodrop^{TM} 8000, and potential presence of contaminants (e.g., proteins and organic solvents) is evaluated with the A260/A280 and A260/A230 ratios. (B) RNA samples are then evaluated for integrity/fragmentation by smear analysis using the TapeStation 2200, and the proportion of RNA above 200nt (DV200) is estimated. Samples with DV200 >30% are considered adequate. (C) DNA fragmentation and potential presence of PCR inhibitors is estimated with a QC-PCR. DNA samples are categorized according to the ability to amplify 100 to 400bp fragments; amplification of fragments of 400bp is considered optimal and 200-300bp, adequate. (D) Genotyping sex markers to uncover potential mismatches and/or contamination with the SampleID kit. Circle and arrow show heterozygosity (XY alleles, male).

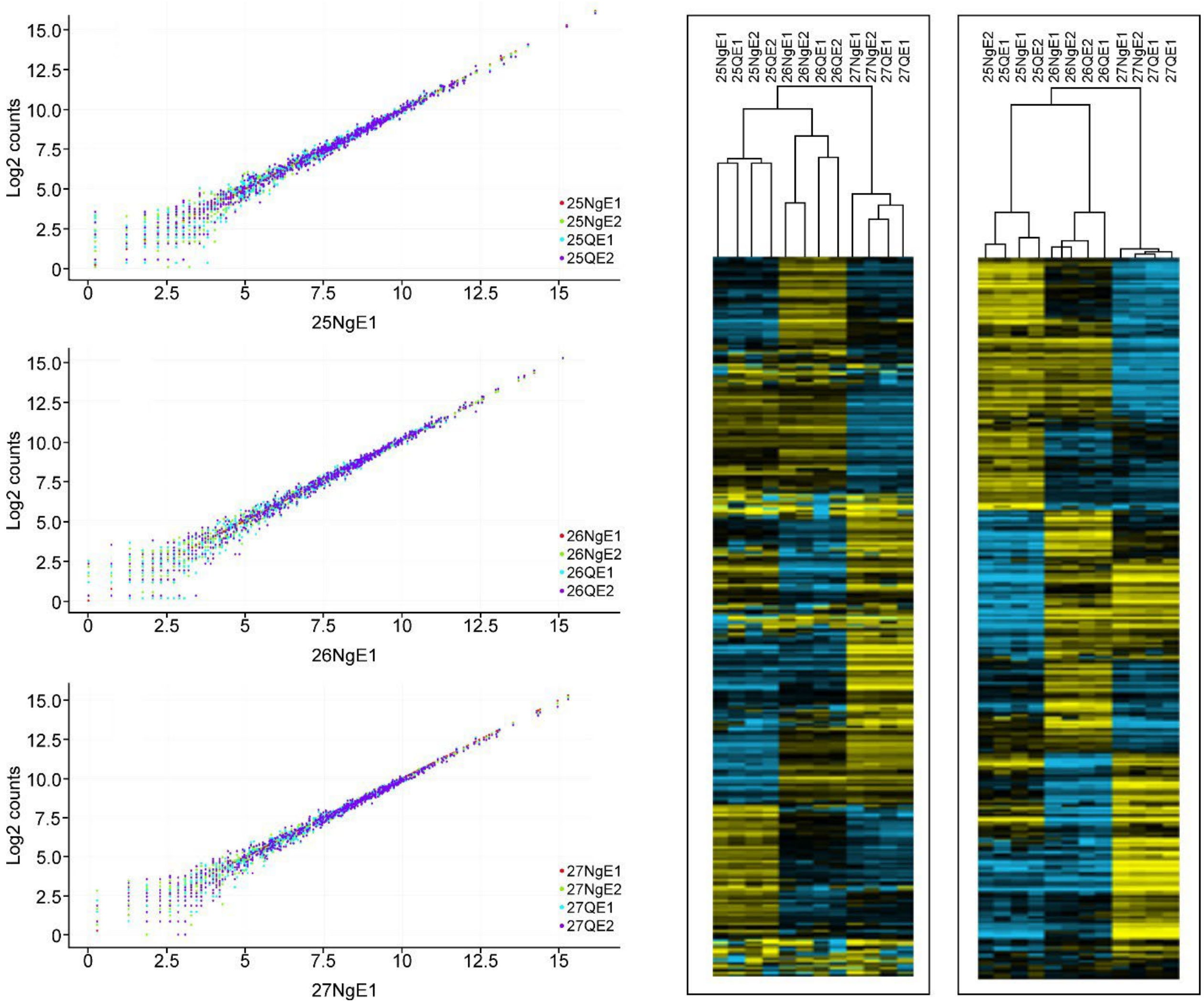

Figure S2. Performance of RNA co-extraction kit and elution on Nanostring. Scatter plots (left) show comparative signals obtained from extractions using two kits (Ng, Q) and two independent RNA elutions (E1, E2) from each of the melanomas # 25, 26, and 27. The scatter occurs for the low intensity points only. Heatmap (right) of Nanostring gene expression normalized data clustered by row and column. First and second eluates rendered RNAs with very similar gene expression for all genes, and after filtering or removal of genes-probes with low signals (far right).

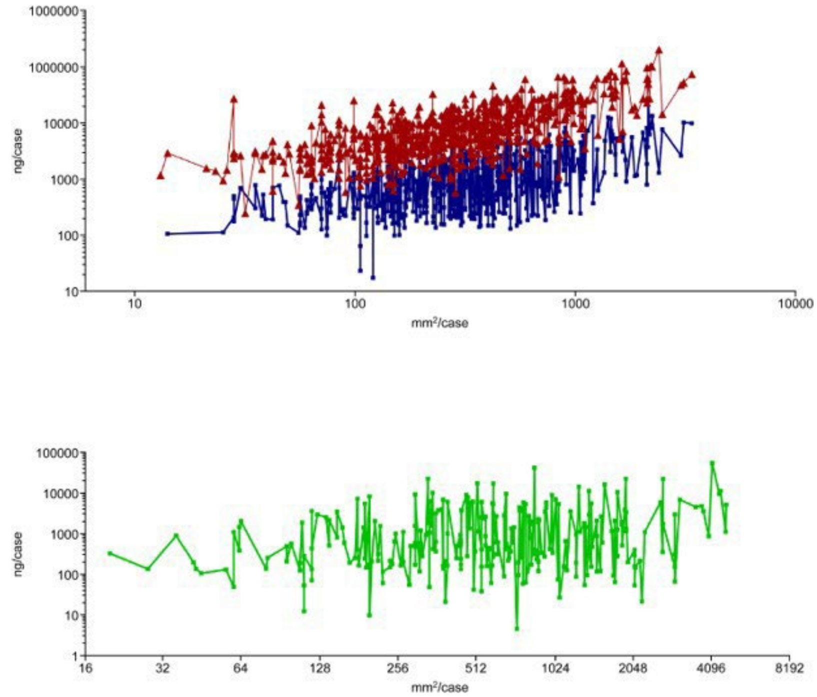

Figure S3. Effect of FFPE tissue input on the yield of co-extracted nucleic acids. Quantity of co-extracted DNA and RNA from tumor (top), and dsDNA from non-tumor (bottom) FFPE tissues, per case and per targeted tissue area. Tumor totRNA is depicted in maroon, tumor dsDNA in blue, and non-tumor dsDNA in green. Correlations between the amount of tumor and non-tumor dsDNA with targeted tissue area were r2= 0.374, and r2= 0.066, respectively.

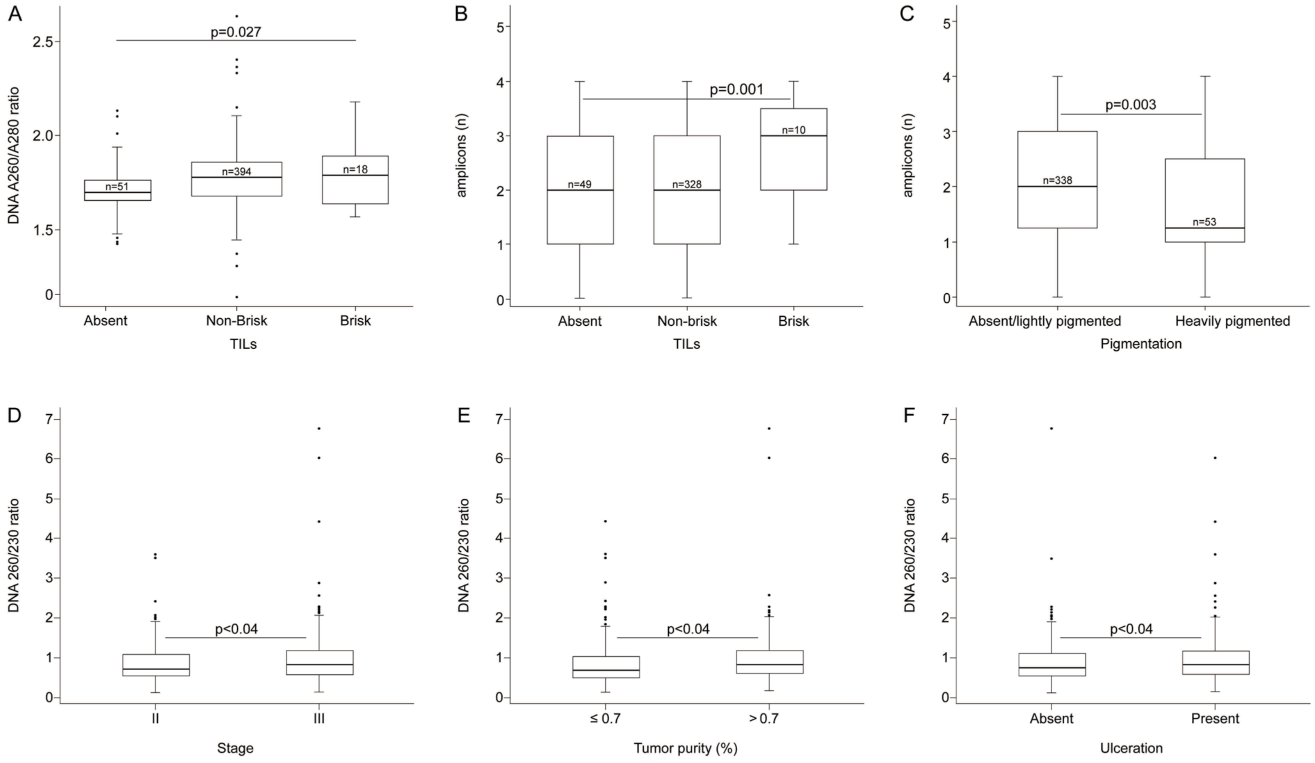

Figure S4. Effect of TILs and pigmentation on DNA purity and amplifiability. TILs, tumor infiltrating lymphocytes; n, number of amplicons ranging from 100 to 400bp.

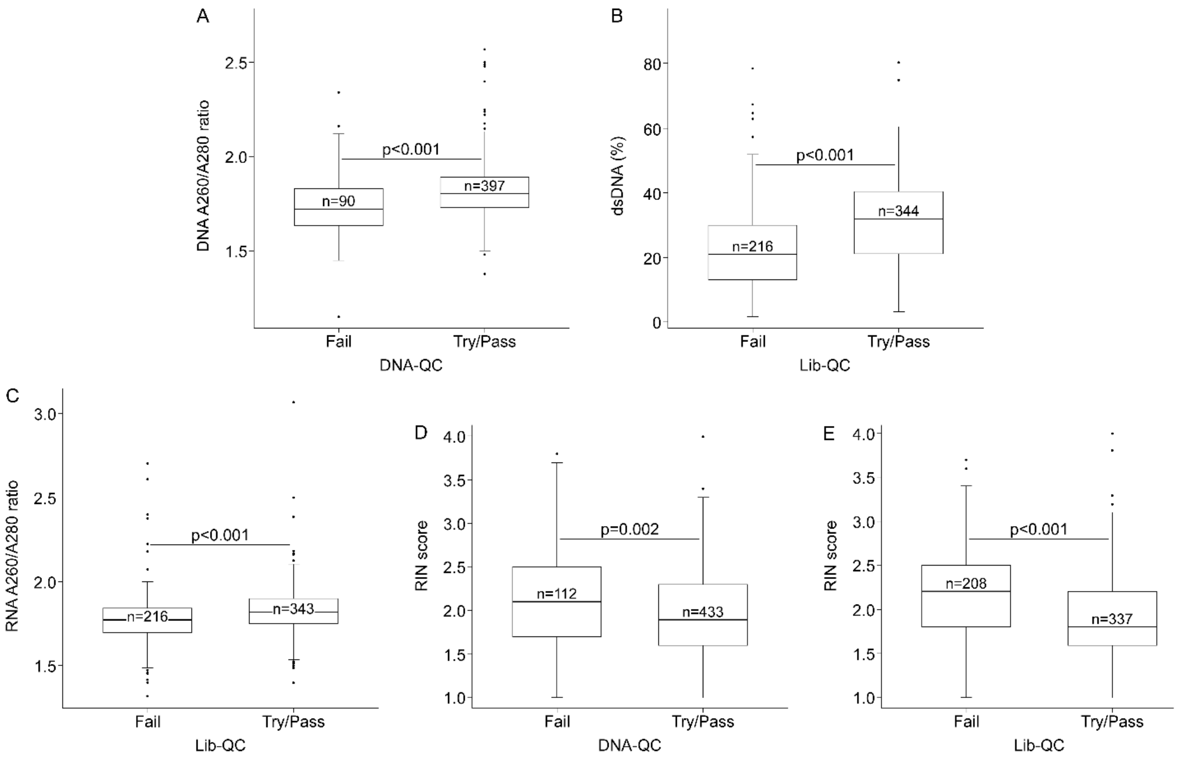

Figure S5. Effect of the DNA and RNA quality on recommendations to proceed with synthesis of libraries and NGS/MSK-IMPACT^{TM}. Recommendation as provided by the genetics core lab (IGO core) to proceed with the synthesis of libraries (A, D), and the MSK-IMPACT^{TM} (B, C, E).

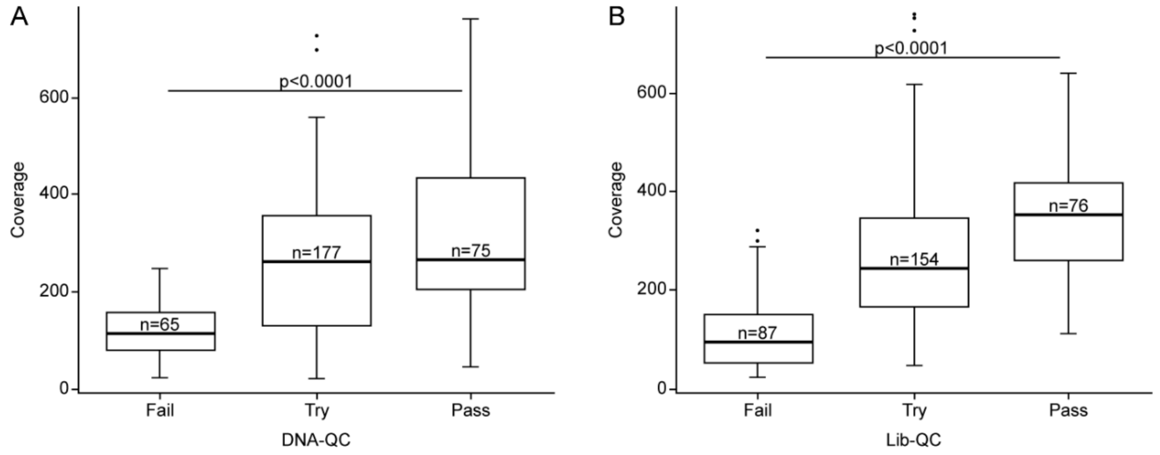

Figure S6. Comparison of DNA- and library-QC recommendations with coverage obtained in melanomas screened with the MSK-IMPACT^{TM} assay. Recommendation as provided by the genetics core lab at MSK (IGO core) to proceed with the synthesis of libraries(A), and the MSK-IMPACT^{TM} (B). Coverage refers to the fold coverage obtained by NGS.
