# Supplementary material for: InterMEL: An international biorepository and clinical database to uncover predictors of survival in early-stage melanoma
Source: PLoS One. 2023 Apr 3;18(4):e0269324. doi: 10.1371/journal.pone.0269324 (PMC10069769; doi:10.1371/journal.pone.0269324)
Supplement: S4 Appendix — (PDF) [file pone.0269324.s005.pdf]

Memorial Sloan Kettering Cancer Center

Oct 17, 2022

Irene Orlow, DSc, MS
Attending Biologist & Laboratory Member
Department of Epidemiology & Biostatistics
Memorial Sloan Kettering Cancer Center

Dear Dr. Orlow,

In support of the InterMEL consortium study, as discussed at the InterMEL meetings, my group developed a statistical method that improves somatic mutation calling in the absence of a matched sample of normal cells. The key insight for accomplishing improved mutation calling in cases with only tumor samples is recognition of the fact that, for variants with normal copy number, germline mutations should produce a variant allele frequency (VAF) of around 50%. While a similar variant allele frequency of 50% would also be expected for somatic mutations in pure tumor samples, typically the tumor purity is much less than 50%, resulting in a considerably lower VAF. Making use of techniques that can simultaneously estimate tumor purity, copy number alterations and VAF from tumor only samples we have developed a method for better distinguishing somatic versus germline variants. Using InterMEL samples, we show the method significantly reduces false positive calls from germline sources compared to standard pipelines. On this subject, we are preparing a manuscript for publication.

Sincerely,

Shen Ronglai

Ronglai Shen, PhD
Memorial Sloan-Kettering Cancer Center

Ronglai, Shen
Attending Biostatistician
Biostatistics service
Department of Epidemiology and Biostatistics
shenr@mskcc.org

485 Lexington Avenue, 2nd Floor, New York, NY, 10017 T 646.888.8243 F 929.321.1516
www.mskcc.org

NCI-designated Comprehensive Cancer Center
